# Supplementary material for: Multi‐dataset identification of innovative feature genes and molecular mechanisms in keratoconus
Source: J Cell Mol Med. 2024 Sep 19;28(18):e70079. doi: 10.1111/jcmm.70079 (PMC11412914; doi:10.1111/jcmm.70079)
Supplement: Supplementary file 2 — Material S2. [file JCMM-28-e70079-s002.zip › 7.GSEA analysis/2.ATOH7/KEGG_CALCIUM_SIGNALING_PATHWAY.html]

Details for gene set KEGG\_CALCIUM\_SIGNALING\_PATHWAY[GSEA]

|  || Dataset | ATOH7.group.cls#h\_versus\_l |
| Phenotype | group.cls#h\_versus\_l |
| Upregulated in class | h |
| GeneSet | KEGG\_CALCIUM\_SIGNALING\_PATHWAY |
| Enrichment Score (ES) | 0.3518775 |
| Normalized Enrichment Score (NES) | 1.3320507 |
| Nominal p-value | 0.03564728 |
| FDR q-value | 0.3243949 |
| FWER p-Value | 0.946 |
Table: GSEA Results Summary

  

Fig 1: Enrichment plot: KEGG\_CALCIUM\_SIGNALING\_PATHWAY      
 Profile of the Running ES Score & Positions of GeneSet Members on the Rank Ordered List

  

| SYMBOL | TITLE | RANK IN GENE LIST | RANK METRIC SCORE | RUNNING ES | CORE ENRICHMENT || 1 | NOS1 | na | 0 | 1.649 | 0.0526 | Yes |
| 2 | NOS2 | na | 363 | 0.597 | 0.0611 | Yes |
| 3 | PDE1B | na | 538 | 0.537 | 0.0732 | Yes |
| 4 | CACNA1G | na | 646 | 0.512 | 0.0864 | Yes |
| 5 | CACNA1B | na | 672 | 0.503 | 0.1017 | Yes |
| 6 | PLCE1 | na | 730 | 0.493 | 0.1158 | Yes |
| 7 | PRKACB | na | 829 | 0.474 | 0.1280 | Yes |
| 8 | GNA14 | na | 1038 | 0.442 | 0.1361 | Yes |
| 9 | SLC8A1 | na | 1100 | 0.432 | 0.1481 | Yes |
| 10 | P2RX1 | na | 1113 | 0.430 | 0.1615 | Yes |
| 11 | P2RX7 | na | 1250 | 0.414 | 0.1707 | Yes |
| 12 | CYSLTR1 | na | 1343 | 0.403 | 0.1809 | Yes |
| 13 | PTGER1 | na | 1351 | 0.403 | 0.1935 | Yes |
| 14 | CD38 | na | 1492 | 0.387 | 0.2018 | Yes |
| 15 | CACNA1E | na | 1688 | 0.370 | 0.2080 | Yes |
| 16 | PLCB1 | na | 1718 | 0.367 | 0.2188 | Yes |
| 17 | PRKACG | na | 1784 | 0.361 | 0.2284 | Yes |
| 18 | PDE1A | na | 1971 | 0.346 | 0.2341 | Yes |
| 19 | ADRA1B | na | 2080 | 0.338 | 0.2417 | Yes |
| 20 | ERBB4 | na | 2108 | 0.336 | 0.2517 | Yes |
| 21 | PRKCB | na | 2281 | 0.324 | 0.2570 | Yes |
| 22 | CAMK2D | na | 2442 | 0.313 | 0.2624 | Yes |
| 23 | EDNRA | na | 2486 | 0.310 | 0.2710 | Yes |
| 24 | GRM5 | na | 2585 | 0.304 | 0.2779 | Yes |
| 25 | CHRNA7 | na | 2982 | 0.282 | 0.2754 | Yes |
| 26 | CHRM1 | na | 3002 | 0.281 | 0.2838 | Yes |
| 27 | GRM1 | na | 3125 | 0.274 | 0.2890 | Yes |
| 28 | ATP2B2 | na | 3181 | 0.271 | 0.2961 | Yes |
| 29 | LHCGR | na | 3192 | 0.271 | 0.3044 | Yes |
| 30 | GRIN2C | na | 3488 | 0.256 | 0.3040 | Yes |
| 31 | CALML6 | na | 3497 | 0.256 | 0.3120 | Yes |
| 32 | P2RX5 | na | 3862 | 0.242 | 0.3091 | Yes |
| 33 | ADORA2B | na | 3930 | 0.239 | 0.3148 | Yes |
| 34 | ADRB3 | na | 4154 | 0.231 | 0.3157 | Yes |
| 35 | AVPR1A | na | 4160 | 0.231 | 0.3229 | Yes |
| 36 | HTR2A | na | 4344 | 0.224 | 0.3247 | Yes |
| 37 | AGTR1 | na | 4501 | 0.218 | 0.3272 | Yes |
| 38 | TNNC2 | na | 4579 | 0.216 | 0.3318 | Yes |
| 39 | CAMK4 | na | 4659 | 0.212 | 0.3363 | Yes |
| 40 | RYR2 | na | 4883 | 0.205 | 0.3364 | Yes |
| 41 | ADCY3 | na | 5082 | 0.198 | 0.3370 | Yes |
| 42 | TRPC1 | na | 5314 | 0.191 | 0.3364 | Yes |
| 43 | MYLK2 | na | 5529 | 0.184 | 0.3361 | Yes |
| 44 | PTGFR | na | 5546 | 0.184 | 0.3415 | Yes |
| 45 | ITPKB | na | 5756 | 0.178 | 0.3411 | Yes |
| 46 | ITPKA | na | 5860 | 0.175 | 0.3437 | Yes |
| 47 | PHKG2 | na | 5869 | 0.175 | 0.3490 | Yes |
| 48 | CACNA1H | na | 5961 | 0.172 | 0.3519 | Yes |
| 49 | BST1 | na | 6457 | 0.158 | 0.3426 | No |
| 50 | ADORA2A | na | 6741 | 0.150 | 0.3392 | No |
| 51 | CCKBR | na | 6827 | 0.147 | 0.3414 | No |
| 52 | MYLK3 | na | 7050 | 0.142 | 0.3395 | No |
| 53 | P2RX6 | na | 7086 | 0.141 | 0.3430 | No |
| 54 | PLCD4 | na | 7237 | 0.137 | 0.3430 | No |
| 55 | PPP3CC | na | 7238 | 0.137 | 0.3474 | No |
| 56 | P2RX4 | na | 7253 | 0.137 | 0.3513 | No |
| 57 | ITPR1 | na | 7473 | 0.131 | 0.3492 | No |
| 58 | BDKRB1 | na | 7717 | 0.125 | 0.3461 | No |
| 59 | PRKCA | na | 7938 | 0.120 | 0.3436 | No |
| 60 | GRPR | na | 8199 | 0.114 | 0.3397 | No |
| 61 | SLC8A3 | na | 8291 | 0.113 | 0.3407 | No |
| 62 | CACNA1A | na | 8517 | 0.108 | 0.3376 | No |
| 63 | CACNA1S | na | 8751 | 0.103 | 0.3342 | No |
| 64 | P2RX3 | na | 8827 | 0.102 | 0.3352 | No |
| 65 | GRIN1 | na | 8945 | 0.100 | 0.3350 | No |
| 66 | HRH1 | na | 9263 | 0.094 | 0.3289 | No |
| 67 | SLC25A4 | na | 9319 | 0.093 | 0.3303 | No |
| 68 | SLC25A31 | na | 9626 | 0.088 | 0.3242 | No |
| 69 | SPHK2 | na | 9717 | 0.086 | 0.3243 | No |
| 70 | TBXA2R | na | 10053 | 0.080 | 0.3172 | No |
| 71 | CALM3 | na | 10070 | 0.080 | 0.3193 | No |
| 72 | ADCY8 | na | 10448 | 0.075 | 0.3107 | No |
| 73 | RYR1 | na | 10489 | 0.074 | 0.3119 | No |
| 74 | GNAL | na | 11321 | 0.061 | 0.2899 | No |
| 75 | VDAC1 | na | 11345 | 0.061 | 0.2911 | No |
| 76 | ADRA1A | na | 11372 | 0.061 | 0.2923 | No |
| 77 | NOS3 | na | 11409 | 0.060 | 0.2932 | No |
| 78 | PPP3CA | na | 11626 | 0.057 | 0.2888 | No |
| 79 | PHKA2 | na | 11792 | 0.055 | 0.2858 | No |
| 80 | PPP3CB | na | 11848 | 0.055 | 0.2859 | No |
| 81 | P2RX2 | na | 12344 | 0.048 | 0.2731 | No |
| 82 | PRKACA | na | 12347 | 0.048 | 0.2746 | No |
| 83 | EGFR | na | 12485 | 0.046 | 0.2721 | No |
| 84 | PLCZ1 | na | 12549 | 0.045 | 0.2717 | No |
| 85 | PRKX | na | 12697 | 0.044 | 0.2689 | No |
| 86 | ATP2B4 | na | 12874 | 0.042 | 0.2651 | No |
| 87 | TNNC1 | na | 13253 | 0.038 | 0.2554 | No |
| 88 | ATP2A3 | na | 13569 | 0.035 | 0.2473 | No |
| 89 | SLC25A6 | na | 13704 | 0.033 | 0.2445 | No |
| 90 | ATP2B3 | na | 13948 | 0.031 | 0.2385 | No |
| 91 | HRH2 | na | 14053 | 0.030 | 0.2364 | No |
| 92 | TACR2 | na | 14124 | 0.029 | 0.2353 | No |
| 93 | GNAS | na | 14632 | 0.024 | 0.2214 | No |
| 94 | CHP1 | na | 14674 | 0.024 | 0.2210 | No |
| 95 | HTR7 | na | 15397 | 0.018 | 0.2007 | No |
| 96 | PPP3R1 | na | 15411 | 0.018 | 0.2009 | No |
| 97 | PPID | na | 15617 | 0.016 | 0.1954 | No |
| 98 | EDNRB | na | 15864 | 0.014 | 0.1888 | No |
| 99 | CALM1 | na | 16251 | 0.011 | 0.1779 | No |
| 100 | VDAC2 | na | 16978 | 0.005 | 0.1571 | No |
| 101 | CYSLTR2 | na | 17176 | 0.003 | 0.1515 | No |
| 102 | VDAC3 | na | 17448 | 0.001 | 0.1436 | No |
| 103 | CHRM2 | na | 17526 | -0.000 | 0.1414 | No |
| 104 | ADRB1 | na | 17706 | -0.002 | 0.1363 | No |
| 105 | LTB4R2 | na | 17948 | -0.004 | 0.1294 | No |
| 106 | SLC25A5 | na | 18293 | -0.007 | 0.1197 | No |
| 107 | GNA11 | na | 18372 | -0.007 | 0.1176 | No |
| 108 | ITPR3 | na | 18764 | -0.011 | 0.1067 | No |
| 109 | PHKA1 | na | 18871 | -0.012 | 0.1040 | No |
| 110 | OXTR | na | 19160 | -0.014 | 0.0961 | No |
| 111 | ERBB3 | na | 19354 | -0.016 | 0.0910 | No |
| 112 | HTR2B | na | 19789 | -0.020 | 0.0791 | No |
| 113 | PLCD1 | na | 19875 | -0.021 | 0.0773 | No |
| 114 | PTK2B | na | 20285 | -0.025 | 0.0662 | No |
| 115 | CALML3 | na | 20596 | -0.028 | 0.0581 | No |
| 116 | GNAQ | na | 20613 | -0.028 | 0.0586 | No |
| 117 | NTSR1 | na | 20678 | -0.028 | 0.0576 | No |
| 118 | ADCY9 | na | 20701 | -0.029 | 0.0579 | No |
| 119 | PLCB4 | na | 20753 | -0.029 | 0.0573 | No |
| 120 | CHRM3 | na | 21000 | -0.032 | 0.0512 | No |
| 121 | PTAFR | na | 21241 | -0.034 | 0.0454 | No |
| 122 | PHKB | na | 21278 | -0.035 | 0.0454 | No |
| 123 | ADCY2 | na | 21650 | -0.039 | 0.0359 | No |
| 124 | CALM2 | na | 21949 | -0.042 | 0.0287 | No |
| 125 | CACNA1C | na | 22079 | -0.044 | 0.0263 | No |
| 126 | PLCD3 | na | 22176 | -0.045 | 0.0250 | No |
| 127 | CAMK2A | na | 22272 | -0.046 | 0.0237 | No |
| 128 | MYLK | na | 22661 | -0.050 | 0.0141 | No |
| 129 | SPHK1 | na | 23089 | -0.056 | 0.0035 | No |
| 130 | CAMK2G | na | 23214 | -0.058 | 0.0017 | No |
| 131 | ADRB2 | na | 23374 | -0.060 | -0.0009 | No |
| 132 | ATP2A2 | na | 23505 | -0.062 | -0.0027 | No |
| 133 | SLC8A2 | na | 23552 | -0.063 | -0.0021 | No |
| 134 | ERBB2 | na | 24092 | -0.071 | -0.0154 | No |
| 135 | PLCB3 | na | 24121 | -0.071 | -0.0139 | No |
| 136 | PLCG2 | na | 24577 | -0.078 | -0.0246 | No |
| 137 | ATP2B1 | na | 24793 | -0.082 | -0.0282 | No |
| 138 | GRIN2D | na | 25022 | -0.086 | -0.0321 | No |
| 139 | PLCG1 | na | 25238 | -0.090 | -0.0354 | No |
| 140 | CHRM5 | na | 25250 | -0.090 | -0.0329 | No |
| 141 | CCKAR | na | 25994 | -0.104 | -0.0511 | No |
| 142 | PDE1C | na | 26063 | -0.105 | -0.0497 | No |
| 143 | TACR1 | na | 26193 | -0.108 | -0.0500 | No |
| 144 | CACNA1D | na | 26374 | -0.112 | -0.0516 | No |
| 145 | PHKG1 | na | 26386 | -0.112 | -0.0484 | No |
| 146 | ADCY7 | na | 26708 | -0.119 | -0.0539 | No |
| 147 | ITPR2 | na | 26843 | -0.122 | -0.0539 | No |
| 148 | CACNA1F | na | 27070 | -0.127 | -0.0563 | No |
| 149 | DRD1 | na | 27257 | -0.132 | -0.0575 | No |
| 150 | ADCY4 | na | 27391 | -0.136 | -0.0570 | No |
| 151 | HTR5A | na | 27915 | -0.149 | -0.0674 | No |
| 152 | PLCB2 | na | 28336 | -0.160 | -0.0745 | No |
| 153 | DRD5 | na | 28427 | -0.163 | -0.0719 | No |
| 154 | GRIN2A | na | 28440 | -0.163 | -0.0671 | No |
| 155 | CHP2 | na | 28497 | -0.164 | -0.0634 | No |
| 156 | AVPR1B | na | 28730 | -0.171 | -0.0647 | No |
| 157 | PRKCG | na | 28978 | -0.178 | -0.0662 | No |
| 158 | ATP2A1 | na | 29423 | -0.192 | -0.0729 | No |
| 159 | F2R | na | 29759 | -0.202 | -0.0762 | No |
| 160 | CALML5 | na | 29931 | -0.208 | -0.0745 | No |
| 161 | GNA15 | na | 30207 | -0.218 | -0.0755 | No |
| 162 | PTGER3 | na | 30618 | -0.233 | -0.0799 | No |
| 163 | BDKRB2 | na | 30702 | -0.236 | -0.0748 | No |
| 164 | TACR3 | na | 30811 | -0.240 | -0.0703 | No |
| 165 | ADCY1 | na | 30908 | -0.244 | -0.0653 | No |
| 166 | HTR2C | na | 30965 | -0.246 | -0.0591 | No |
| 167 | PLN | na | 31826 | -0.284 | -0.0749 | No |
| 168 | CACNA1I | na | 32230 | -0.308 | -0.0767 | No |
| 169 | ADRA1D | na | 32355 | -0.317 | -0.0702 | No |
| 170 | PPP3R2 | na | 32636 | -0.337 | -0.0676 | No |
| 171 | PDGFRB | na | 32781 | -0.349 | -0.0606 | No |
| 172 | RYR3 | na | 33675 | -0.436 | -0.0726 | No |
| 173 | PDGFRA | na | 33905 | -0.477 | -0.0640 | No |
| 174 | HTR6 | na | 34125 | -0.524 | -0.0536 | No |
| 175 | HTR4 | na | 34323 | -0.581 | -0.0408 | No |
| 176 | TRHR | na | 34469 | -0.658 | -0.0241 | No |
| 177 | CAMK2B | na | 34693 | -0.989 | 0.0010 | No |
Table: GSEA details [plain text format]

  

Fig 2: KEGG\_CALCIUM\_SIGNALING\_PATHWAY      
 Blue-Pink O' Gram in the Space of the Analyzed GeneSet

  

Fig 3: KEGG\_CALCIUM\_SIGNALING\_PATHWAY: Random ES distribution      
 Gene set null distribution of ES for **KEGG\_CALCIUM\_SIGNALING\_PATHWAY**
